# Size-Dependent Electrochemical Response and Dopamine Sensitivity of Aptamer-Modified Printed Gold Nanoparticle Structures

**DOI:** 10.1021/acsomega.5c13417

**Published:** 2026-05-07

**Authors:** Santhosh Adhinarayanan, Harikrishnan Muraleedharan Jalajamony, Soumadeep De, Renny Edwin Fernandez

**Affiliations:** 1 Department of Engineering, 6041 Norfolk State University, Norfolk, Virginia 23504, United States; 2 Department of Materials Science and Engineering, 6041 Norfolk State University, Norfolk, Virginia 23504, United States

## Abstract

We report a comparative evaluation of dopamine (DA) sensing using gold nanoparticle (AuNP)-based electrodes fabricated from precursor-free aqueous suspension through atmospheric plasma-assisted printing. In this study, electrodes printed from 20, 40, and 80 nm AuNPs in aqueous suspension were analyzed to establish correlations between nanoparticle size, surface morphology, and dopamine redox performance. Morphological and optical characterization of the printed structures confirmed the preserved nanoscale morphology and stable electrochemical behavior. Electrochemical analysis revealed stable, size-dependent redox behavior for the printed electrodes and long-term stability over 100 cycles. Electrodes printed from smaller nanoparticles (20 nm) exhibited reduced Δ*E* values and faster electron-transfer kinetics, while intermediate-sized electrodes (40 nm) offered the most balanced combination of electroactive surface area, morphological stability, and charge-transfer efficiency. In contrast, larger nanoparticle-derived electrodes (80 nm) had lower surface area, resulting in diminished redox reversibility and sensitivity. Aptamer functionalization followed by thiol backfilling enhanced dopamine selectivity and sensitivity across 1–100 μM, achieving micromolar detection limits and excellent reproducibility. This clean, scalable approach provides a robust foundation for printing low-cost, flexible biosensors capable of selective detection and future integration into wearable diagnostic platforms.

## Graphical abstract

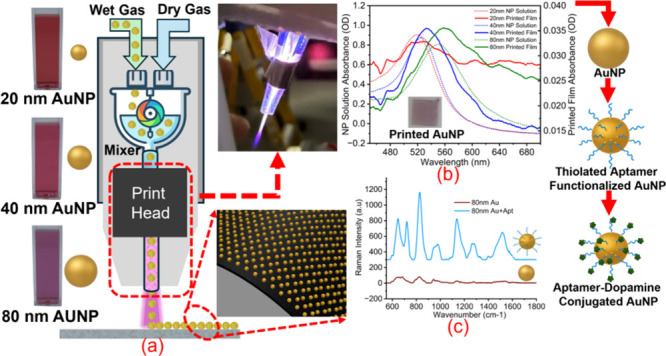

## Introduction

In healthy individuals, dopamine concentrations are typically about 20 ng mL^–1^ in plasma,^1,2^ ∼18 pg mL^–1^ in saliva,^3^ and 0.2–1 mg mL^–1^ in urine.^4^ Rapid detection of dopamine within these ranges is clinically vital for biomedical diagnosis. Dopamine (DA) often coexists with ascorbic acid (AA) and uric acid (UA) in biological fluids,^5,6^ prompting the development of analytical methods that enable selective and simultaneous determination of these species despite their overlapping oxidation potentials.

Gold-based dopamine sensing has been extensively researched.^7–10^ Numerous colorimetric and electrochemical sensing systems have been developed.^11–14^ In colorimetric assays, gold nanoparticles (AuNPs) undergo dopamine-induced aggregation, resulting in distinct optical changes that facilitate naked-eye detection and spectrophotometric quantification with nanomolar.^15^ Various strategies have been reported to enhance selectivity against common interferents such as AA and UA. For instance, AuNP probes functionalized with molecular recognition ligands^16,17^ enable aggregation-based detection with submicromolar limits of detection, validated in biological samples. Cyclodextrin-modified AuNP assemblies provided ordered aggregation, yielding an ultralow limit of detection (LOD) of ∼3 nM, two well-defined linear response ranges (20–250 nM and 350–1600 nM), and excellent colloidal stability for 30 days at 4 °C.^18^ Copper ion-assisted dopamine-induced AuNP aggregation generates colorimetric signals proportional to DA.^19^ Aptamer-based systems further enhanced the sensors: AuNPs stabilized by dopamine-binding aptamers enabled rapid, label-free detection with sub-μM sensitivity,^20,21^ while dual-mode aptamer–AuNP systems combining colorimetric and fluorescence detection achieved submicromolar sensitivity for dopamine, with LODs of ∼0.14 μM (colorimetric) and ∼0.0787 μM (fluorometric).^22^ Thioglycolic acid (TGA^2–^)- assisted AuNP aggregation reaches naked-eye detection limits of ∼10^–7^ M and demonstrates selectivity in complex matrices, such as urine and serum.^23^

Electrochemical platforms leveraging nanostructured carbons, metal oxides, conducting polymers, and micro/nanoengineered gold electrodes have delivered robust dopamine sensing with high selectivity versus AA/UA. MoS_2_/rGO-modified glassy carbon electrodes (GCEs) resolve AA/DA/UA simultaneously with well-separated differential pulse voltammetry (DPV) peaks at – 64 mV, 168 mV, and 320 mV (vs Ag/AgCl), achieving a DA LOD of 0.05 μM.^24^ Graphene/PEI/AuNP composite exhibits a DA redox near +0.20 V, a 2–48 μM linear range, LOD 0.2 μM, and validation in urine.^25^ PANI–metal-oxide nanocomposites (PANI–NiO/ZnO/Fe_3_O_4_@GCE) achieve nanomolar level detection with strong selectivity against AA and serotonin.^26^ Au nanoelectrodes have been reported to electrochemically sense 1–100 μM DA.^27^

GO/AuNP/GO sandwiches on ITO yield electrochemical responses in the 0.1–30 μM detection range with LOD of ∼ 1.28 μM and resilience to AA and glucose interference.^28^ PEDOT multilayers decorated with AuNPs expand AA–DA potential separation, underscoring the role of Au decoration in facilitating fast charge transfer.^29,30^ Graphene-AuNP films on GCE enabled DA detection in serum samples with a LOD of ∼ 1.86 μM^31^ while green-synthesized GA-RGO/AuNP@GCE achieves a 2.6 nM LOD.^32^ Pt microelectrode (100 μm) coated with rGO/AuNP enables in vivo striatal DA monitoring with a 16.57 nM LOD, highlighting the stability and physiological relevance of Au-based electrodes.^33^ CYST–SAM/Au nanoparticle conjugates exhibited enhanced electron-transfer kinetics, achieving dopamine detection limits of 0.13–0.22 μM and illustrating the intrinsic “electron-antenna” behavior of nanoscale gold surfaces.^34^ Collectively, these studies demonstrate that combining high-surface-area structures with AuNP enhances the Au-enabled catalysis, yielding selective DA readouts near +0.2 to 0.3 V, spanning nM−μM concentrations, and validated across serum and urine.

Direct nanoparticle printing enables the fabrication of electrochemical sensors with controlled surface area and morphology. Although selecting AuNP sizes (20, 40, 80 nm) offers tunability, inkjet printing typically involves binders/surfactants and combined with inkjet fluidics, poses challenges in microscale structural control and baseline reproducibility. Plasma-aided printing addresses this by depositing binder-free nanoparticle aerosols under low-temperature plasma, where rapid solvent removal occurs simultaneously with substrate surface activation that creates nanoscale pores and active sites, forming a foundation layer for strong mechanical and chemical anchoring. This plasma-induced anchoring is particularly important for electrochemical applications, where loosely bound nanoparticles can detach during repeated measurements. The process is further optimized to remain compatible with low-temperature commercial TE100 screen-printed electrodes without causing substrate deformation, enabling practical use on disposable platforms.

We recently reported the direct printing of stable metal-oxide microelectrodes combining wearable-grade mechanical endurance with electrochemical stability for real-time pH and H_2_O_2_ sensing under biologically relevant conditions.^35,36^ Building on this approach, the present work prints AuNPs from stable aqueous suspensions to fabricate reproducible dopamine sensors, systematically correlates nanoparticle size and plasma conditions with layer morphology and electron-transfer behavior, and integrates thiolated dopamine aptamer functionalization with MCH backfilling to enhance sensitivity, selectivity, and linear sensing range.

## Materials and Methods

### Materials and Reagents

All chemicals used in this study were of analytical grade. Standard citrate-stabilized spherical gold nanoparticles (20, 40, and 80 nm) were purchased from CytoDiagnostics. Dopamine hydrochloride (DA), uric acid (UA), ascorbic acid (AA), potassium ferricyanide (K_3_[Fe­(CN)_6_]), potassium chloride (KCl), Tris­(hydroxymethyl)­aminomethane hydrochloride (Tris-HCl), sodium chloride (NaCl), Sodium Phosphate Dibasic (Na_2_HPO_4_), Potassium Phosphate Monobasic (KH_2_PO_4_), magnesium chloride (MgCl_2_), and Sodium phosphate buffer (1 M stock, pH 7.6) were obtained from Sigma-Aldrich and prepared using deionized (DI) water. Dithiothreitol (DTT) and 6-mercaptohexanol (MCH) were sourced from Fisher Scientific. Thiol-terminated DNA aptamers (5′-/Thiol-C6/-ACGACATTACGGGACCTTGCTAAAGGTGGAATTATGTCGT-3′) were purchased from Integrated DNA Technologies (IDT).

### Plasma-Aided Printing of Au Nanoparticle Layers

The gold nanoparticle (AuNP) layer (Figure 1a–c) was fabricated using an atmospheric plasma–assisted inkjet printing system (Space Foundry Inc., USA). Standard citrate-stabilized AuNP colloids (20, 40, and 80 nm) purchased from Cytodiagnostics Inc. were used directly as binder-free, surfactant-free, ready-to-use aqueous suspensions, without any additional solvent or diluent, thereby maintaining deionized water as the sole solvent. Printing was conducted using the low-temperature atmospheric plasma jet operated under a controlled inert gas mixture (95% Ar + 5% H_2_). In this process, the AuNP colloidal suspension is first converted into a fine mist, and the carrier gas is passed through this mist (wet gas), which is then transported to the printhead along with a dry sheath gas. Within the plasma plume, rapid solvent evaporation, nanoparticle activation, and simultaneous substrate surface modification occur, creating nanoscale pores and active sites that serve as a foundation layer for strong mechanical interlocking and adhesion without any postprinting process. This combined action promotes robust nanoparticle anchoring to the substrate and yields uniform, well-adhered AuNP layers (Figure 1c).

**Figure 1:**
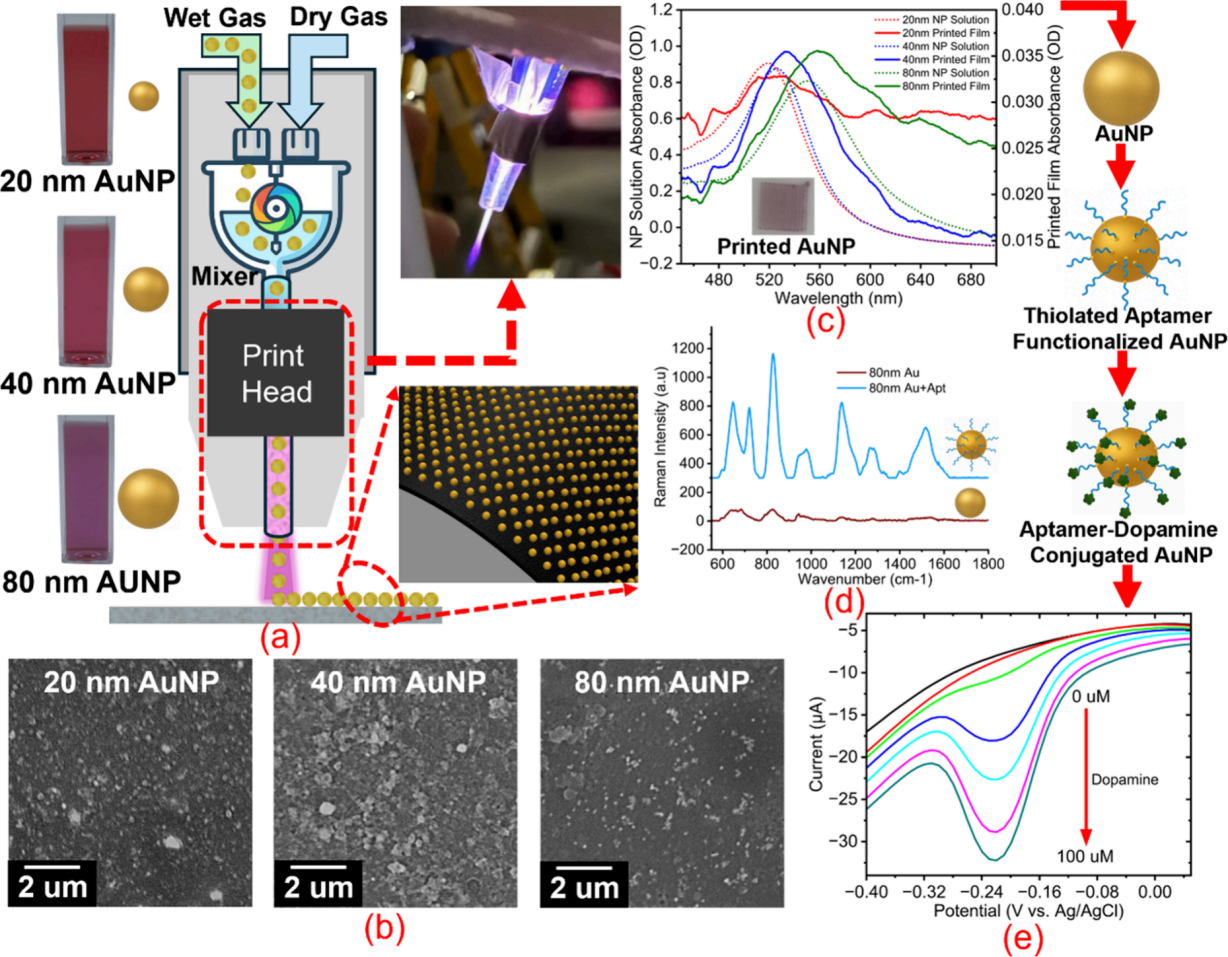
a) Plasma-assisted direct printing of AuNPs (20, 40, and 80 nm) on TE100 screen-printed electrodes, b) SEM images of plasma-printed AuNPs at 18 kV for the three nanoparticle sizes (20 nm, 40 nm, 80 nm), c) UV–Visible spectroscopy comparing the absorbance spectra of AuNP solutions and their corresponding plasma-printed layer spectra for each nanoparticle size, d) Raman spectra of 80 nm AuNPs before and after thiolated aptamer immobilization, highlighting the characteristic aptamer-associated vibrational peaks, e) Differential Pulse Voltammetry (DPV) response of aptamer–dopamine-conjugated AuNP modified electrodes showing increasing current response with dopamine concentration (0–100 μM), demonstrating sensing sensitivity.

An AuNP layer was printed on the working electrode of commercial screen-printed carbon electrodes (SPEs, TE100; three-electrode configuration), for electrochemical sensing. For surface characterization, identical AuNP layers were printed on silicon substrates for SEM (Figure 1b) and on quartz substrates for UV–visible (Figure 1d) and Raman spectroscopy (Figure 1e). Two sets of electrodes were printed at plasma voltages of 14 kV and 18 kV (peak-to-peak) at a frequency of 30 kHz, using a 100% mist level and a print speed of 60 mm/min. The gas flow rates were maintained at 150 SCCM for the wet aerosol and 300 SCCM for the dry sheath gas, with a printhead–substrate distance of approximately 1 mm. All printing parameters were optimized to promote strong adhesion and uniform surface coverage of the nanoparticles while avoiding plasma-induced deformation of the low-temperature SPE substrate.

### Aptamer Reduction and Purification

Thiol-terminated single-stranded DNA (ssDNA) aptamers were reduced with dithiothreitol (DTT) to generate free thiols for immobilization. The lyophilized aptamer was dissolved in deionized (DI) water to prepare a 500 μM stock. A 50 μL aliquot of this stock was mixed with 450 μL of freshly prepared 0.15 M sodium phosphate buffer (pH 8.5) containing 0.10 M DTT and incubated at room temperature for 1–2 h to ensure complete reduction. The reaction mixture was then purified on a pre-equilibrated NAP-5 desalting column (Cytiva) to remove excess DTT. The principal eluate (∼1 mL) provided a purified aptamer solution at ∼25 μM. The exact concentration was determined by UV–Visible absorbance at 260 nm using the sequence-specific extinction coefficient supplied by the manufacturer. The reduced aptamer was then diluted in immobilization buffer to the desired concentration for electrode functionalization.

### Aptamer Immobilization and MCH Backfilling

Aptamer immobilization on gold nanoparticle (AuNP)-modified electrodes was performed using drop-casting. A 50 μL aliquot of 1 μM reduced, thiol-terminated dopamine-specific aptamer dissolved in immobilization buffer (20 mM Tris-HCl, 100 mM NaCl, 2 mM MgCl_2_, pH 7.4) was drop-cast onto the electrode surface and incubated in a humidified closed chamber for 2 h at room temperature to promote covalent Au–S bond formation. Following immobilization, the electrodes were gently rinsed with 0.1 M PBS to remove unbound aptamer molecules. Subsequently, for surface passivation, a 50 μL aliquot of 1 mM 6-mercapto-1-hexanol (MCH) in 0.1 M PBS (pH 7.4) was drop-cast onto the electrode and incubated in a humidified closed chamber for 30 min at room temperature to block residual exposed gold sites and minimize nonspecific adsorption. The electrodes were then gently rinsed with 0.1 M PBS (pH 7.4) and immediately used for subsequent electrochemical measurements.

### Characterization of Plasma-Printed Au Layers and Aptamer-Modified Surfaces

The surface morphology and coverage of plasma-printed Au layers (20, 40, and 80 nm; 14–18 kV) were examined by scanning electron microscopy (SEM; Semplor Nanos) on printed Si substrates. Elemental composition and Au spatial distribution were assessed by energy-dispersive X-ray spectroscopy (EDS). Optical properties were characterized on printed quartz substrates by ultraviolet–visible (UV–Vis) spectroscopy using an Ocean Optics FLAME-S-XR1_ES to identify localized surface plasmon resonance (LSPR) characteristics for each particle size. Raman spectra were acquired on printed silicon substrates before and after aptamer immobilization using a Thunder Optics Affa Raman System TO-ARS-785-TEC-E to verify the presence of aptamer on the Au surface. All measurements were carried out under identical instrumental conditions with consistent sample preparation and handling to ensure reproducibility.

### Electrochemical Measurement Setup

Electrochemical characterization was performed using the plasma-printed layers as working electrodes in a three-electrode cell, with an Ag/AgCl reference electrode (saturated KCl) and a platinum wire counter electrode. All measurements were conducted at room temperature (25 °C) using an EmStat3 potentiostat (PalmSens). Phosphate-buffered saline (PBS) was prepared in-house by dissolving 136.893 mM sodium chloride (NaCl), 2.683 mM potassium chloride (KCl), 10.144 mM sodium phosphate dibasic (Na_2_HPO_4_), and 1.8 mM potassium phosphate monobasic (KH_2_PO_4_) in 1 L of deionized (DI) water, followed by pH adjustment to 7.4 using NaOH and diluted HCl. Required PBS concentrations were obtained by dilution with DI water. Electrode stability was assessed by Cyclic Voltammetry (CV) using 5 mM K_3_[Fe­(CN)_6_] in 0.1 M PBS, with scans initiated at 0.0 V vs Ag/AgCl, swept to +0.5 V, reversed to −0.5 V, and returned to the starting potential, using a step potential of 0.01 V, scan rate of 0.1 V s^–1^, and equilibration time of 1 s. Differential pulse voltammetry (DPV) was performed using 0.5 mM K_3_[Fe­(CN)_6_] to evaluate electrochemical surface properties with scans initiated at +0.5 V vs Ag/AgCl and swept toward −0.8 V vs Ag/AgCl, using a pulse amplitude of 0.10 V, pulse width of 0.02 s, step potential of 0.01 V, scan rate of 0.025 V s^–1^, and equilibration time of 1 s. Dopamine sensing performance was investigated by immersing the electrodes in PBS containing dopamine at concentrations ranging from 1 to 100 μM. Selectivity was examined by introducing ascorbic acid and uric acid at physiologically relevant concentrations to simulate common interfering species.

### Dopamine Sensitivity, Stability, and Selectivity Testing

Sensitivity was assessed by recording DPV responses in PBS over increasing dopamine (DA) concentrations (1–100 μM). For each concentration, DPV was recorded after a 2 min stabilization period. Sensitivity was calculated from the slope of the peak current versus DA concentration (reported in mA mM^–1^). Long-term stability of the printed AuNP layers (20, 40, and 80 nm) was evaluated by performing 100 consecutive CV cycles and monitoring changes in peak current and baseline. Selectivity was tested by stepwise introduction of common electroactive interferents (AA and UA) to simulate physiological conditions. After each addition, a 2 min stabilization was allowed before acquiring DPV. The effect of each interferent on the DA response was quantified as the change in peak current (and peak potential) relative to the DA-only condition.

### Statistical Analysis

All experiments were performed in triplicate to ensure reproducibility. Data are reported as mean ± standard deviation (SD). Statistical significance was evaluated using one-way ANOVA, with *p* < 0.05 considered significant. Results for sensitivity and selectivity were compared among electrode types (bare Au, Au + Apt, and Au + Apt + MCH) to assess the impact of aptamer immobilization on dopamine sensing performance.

## Results and Discussion

### Fabrication of Dopamine Sensing Electrodes

Citrate-stabilized Au nanoparticles (20, 40, and 80 nm) were directly deposited onto screen-printed carbon electrodes (SPEs) using low-temperature atmospheric plasma-assisted jet printing (Figure 1a). The plasma promoted rapid solvent removal and mild surface activation, yielding uniform nanoparticle adhesion and strong interfacial contact with the carbon working surface. The plasma voltage was varied from 14 to 18 kV to assess its effect on layer adhesion and reproducibility; 18 kV produced the most uniform and stable Au layers, suitable for electrochemical sensing. The process required no thermal post-treatment or annealing.

Subsequently, a thiol-terminated dopamine aptamer was immobilized (Figure 1d) onto the printed Au layers to impart molecular recognition. After aptamer coupling, electrodes were rinsed with buffer and dried under ambient conditions. Where indicated, electrodes were further backfilled with MCH to passivate unoccupied Au sites and improve signal specificity. The resulting electrodes, denoted as Au, Au + Apt, and Au + Apt + MCH, were rinsed with buffer and dried under ambient conditions. These modified electrodes underwent morphological, spectroscopic, and electrochemical characterization to evaluate their dopamine sensing performance.

### Structural and Morphological Characterization

The morphological characteristics of the plasma-printed gold nanoparticle (AuNP) layer were examined using SEM, and the elemental composition was confirmed using EDS (Figure 2a–e). A clear dependence of surface morphology on both applied plasma voltage and nanoparticle size was observed.

**Figure 2:**
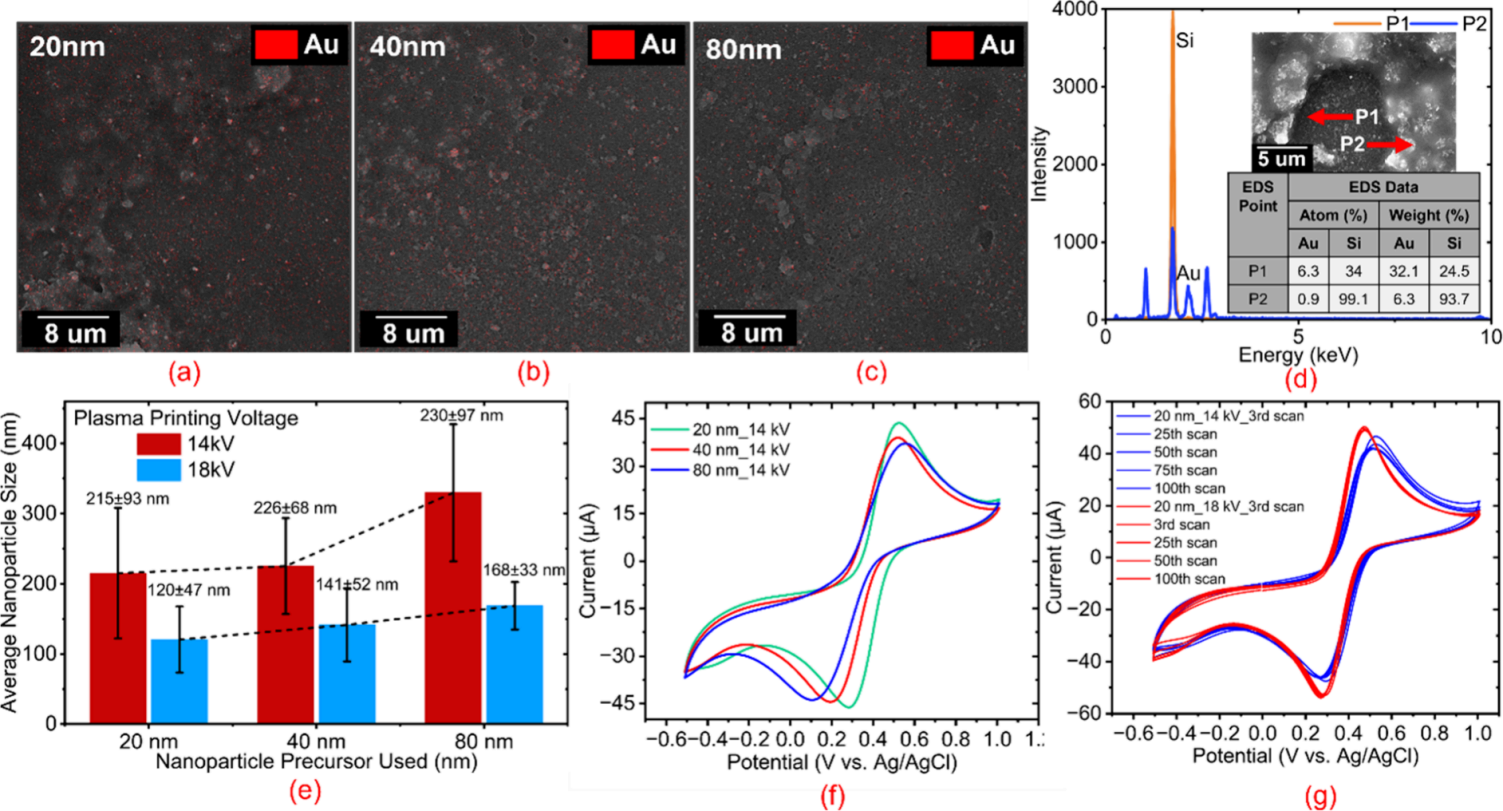
SEM and EDS characterization of plasma-printed AuNP layers and their corresponding electrochemical performance. (a–c) EDS elemental mapping (SEM at 18 kV) showing the spatial distribution of gold (Au) for electrodes printed using 20, 40, and 80 nm AuNP suspension, d) EDS elemental composition of 20 nm AuNP printed at 14 kV, confirming the presence of AuNP in the cluster and Si substrate, e) CV response of 20, 40, and 80 nm AuNP electrodes printed at 14 kV, f) Cycling stability of 20 nm AuNP printed at 14 kV (blue) and 18 kV (red), shown at the third, 25th, 50th, 75th, and 100th cycles, g) Plot of the average Au nanoparticle/cluster size for 20, 40, and 80 nm suspension printed at 14 kV and 18 kV.

At the lower printing voltage (14 kV), all samples exhibited large, agglomerated clusters formed by AuNPs combined with NaCl residues originating from the commercial aqueous solution. These clusters appeared bulky and irregular, indicating insufficient plasma energy to fully activate the surface and effectively separate the nanoparticles during deposition. As a result, the nanoparticles fused into larger aggregates, producing thicker, coarse, impurity-rich cluster networks on the electrode surface (Figure S1a–c). Corresponding EDS elemental mapping of these regions confirmed the presence of Au within the cluster domains, verifying that the agglomerated features observed in SEM are composed of gold nanoparticles (Figure S2a–c). SEM analysis showed average cluster dimensions of 215.1 ± 93.2 nm, 225.2 ± 68.4 nm, and 329.8 ± 97.6 nm for the 20, 40, and 80 nm suspension, respectively, confirming that the lower plasma energy promoted secondary particle fusion and agglomeration regardless of the initial nanoparticle size (Figure 2e).

In contrast, samples printed at the higher voltage (18 kV) exhibited significantly reduced impurities and much finer nanoparticle distributions. The higher plasma energy improved solvent removal and enhanced particle–surface adhesion, reducing NaCl retention and suppressing large-scale clustering. As a result, the layers were thinner, cleaner, and more homogeneously distributed, with smaller nanoparticles and smaller nanoclusters forming a continuous and uniform coating (Figure S3a–c). EDS elemental mapping of the 18 kV printed layers confirmed the presence of Au across the surface; however, the Au signal intensity appeared lower than in the 14 kV samples due to the nanoparticles being smaller and more uniformly dispersed, rather than concentrated into dense clusters (Figure 2a–c). The average particle sizes were 120.3 ± 47.1 nm, 141.5 ± 52.2 nm, and 168.7 ± 33.7 nm for the 20, 40, and 80 nm suspension, respectively (Figure 2e). Notably, the 18 kV/80 nm samples displayed a higher population of small, well-dispersed Au nanoparticles than nanoparticle clusters. The 20 and 40 nm 18 kV samples also contained numerous nanoparticles, forming smaller, tightly packed clusters rather than the large, fused domains observed at 14 kV. These layers maintained a more uniform and continuous surface coverage compared to their 14 kV counterparts, demonstrating that increased plasma energy effectively limited particle fusion and reduced impurity-induced agglomeration.

EDS analysis of the 20 nm AuNP sample printed at 14 kV revealed distinct elemental differences between the substrate and the nanoparticle cluster regions (Figure 2d). The P1 spectrum taken from an exposed Si substrate region showed a dominant Si peak with only a very small trace of Au, indicating minimal particle deposition outside the clustered areas. In contrast, the P2 spectrum acquired from a nanoparticle cluster displayed strong Au peaks together with Na and Cl signals, confirming that the clusters consist of AuNPs coagglomerated with residual NaCl stabilizer from the aqueous solution. This confirms that the large agglomerates observed in SEM originate from both nanoparticle fusion and salt retention. The presence of Na and Cl is characteristic of the lower plasma energy at 14 kV, which is insufficient to fully remove stabilizer residues and separate individual nanoparticles during deposition.

### Size-Dependent CV Response and Cycling Stability of AuNP-Modified Electrodes

As shown in Figure 2f, the cyclic voltammetry (CV) profiles of electrodes printed, revealing a clear size-dependent variation in the reduction peak behavior. The oxidation peak potential remained nearly constant at ∼ 0.50 V across all sizes, while the reduction peak shifted from 0.279 V (20 nm) to 0.189 V (40 nm) and 0.109 V (80 nm), increasing the ΔE from 0.259 V (20 nm) to 0.329 V (40 nm) and 0.439 V (80 nm). The smaller ΔE for 20 nm electrodes indicates faster electron-transfer kinetics and higher redox reversibility, whereas the larger ΔE for 80 nm reflects slower charge transfer due to coarser microstructure. The electroactive surface areas (ECSA), calculated using the Randles–Ševčík equation:

$$I_{p}=2.69\times 10^{5}n^{3/2}AD^{1/2}Cv^{1/2}$$

where Ip is the anodic peak current (A), n is the number of electrons transferred (1 for [Fe­(CN)_6_]), A is the electroactive area (cm^2^), D is the diffusion coefficient (7.6 × 10^–6^ cm^2^ s^–1^), C is the concentration (5 × 10^–6^ mol cm^–3^), and v is the scan rate (0.1 V s^–1^), were 0.037 cm^2^ (20 nm), 0.033 cm^2^ (40 nm), and 0.032 cm^2^ (80 nm) at 14 kV, following the order E20 > E40 > E80. The higher ECSA for smaller nanoparticles confirms that finer nanostructures provide larger electroactive interfaces and promote faster electron transfer, aligning with ΔE trends and SEM results.

The Electrochemical Stability of the printed structures was assessed over 100 consecutive CV cycles to confirm the stability of the surfaces and their usability as a continuous sensing surface. It was evident that the structures printed at higher voltages (18 kV) were extremely stable; the peaks and current remained stable and steady for the full 100 cycles (Figure 2g). Whereas the structures printed at lower voltages (14 kV) showed pronounced baseline drift, peak evolution over the first 10–30 cycles, and gradual decreases in anodic/cathodic peak currents, indicating inferior stability. In contrast, electrodes printed at 18 kV exhibited excellent reproducibility, with overlapping CV curves from 1 to 100 cycles and negligible shifts in peak potential or current (Figure 2g). This enhanced cycling stability is attributed to stronger plasma–surface interactions, denser grafting, improved layer adhesion, and reduced nanoparticle detachment or reorganization during potential sweeps.

### Plasmonic Response and Size-Dependent Optical Characterization

Au nanoparticles (20, 40, and 80 nm) were printed on quartz at the optimized 18 kV plasma voltage for plasmonic evaluation. Prior to printing, UV–Vis spectra of citrate-stabilized AuNP solutions confirmed size and stability, with absorption maxima (λ_max) at 523 nm (20 nm), 531 nm (40 nm), and 553 nm (80 nm) (Figure S4), consistent with standards for spherical Au.

Postprinting, UV–Vis spectra of the plasma-printed layers exhibited localized surface plasmon resonance (LSPR) peaks at 527 nm (20 nm), 531 nm (40 nm), and 559 nm (80 nm) (Figure 3a). These positions closely matched those of their respective aqueous AuNP solutions, indicating that the printed layers retained the optical distinctiveness associated with each nanoparticle size. The preservation of these size-dependent absorption characteristics confirms that plasma printing maintained the nanoparticles’ plasmonic identity rather than converting them into bulk-like gold layers. Minor peak broadening was observed, which may be due to interparticle coupling and layer densification during deposition. Overall, the results demonstrate that plasma-assisted printing size-resolved AuNP structures while preserving their nanoscale features.

**Figure 3:**
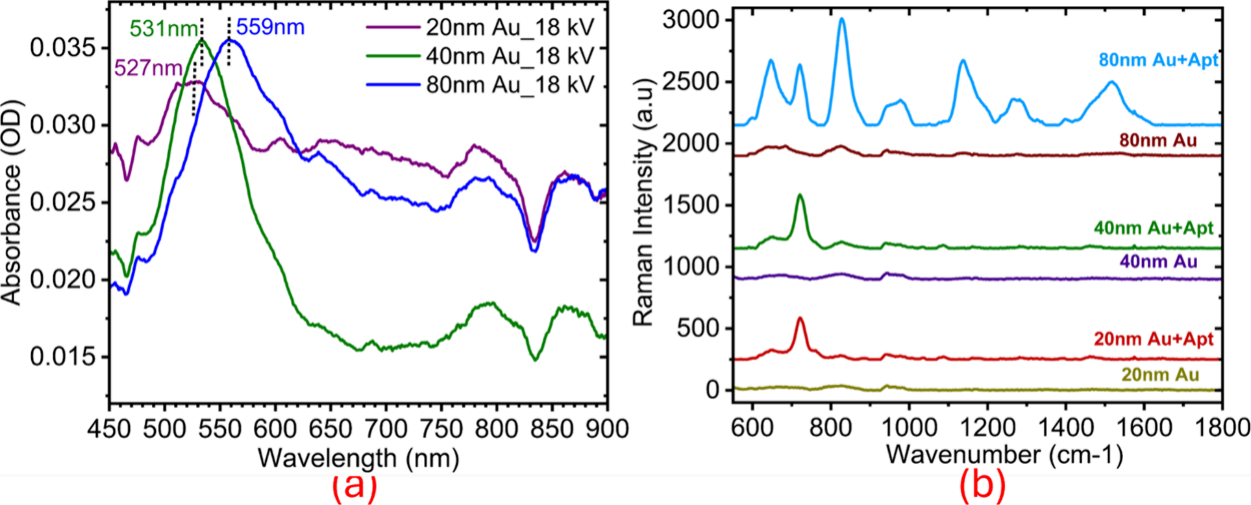
a) UV–Vis Spectra of printed Au layers on quartz (18 kV) displaying nearly identical LSPR peaks, b) Raman/SERS spectra of bare Au, Au + Apt, and Au + Apt + Ag for 20, 40, and 80 nm layers.

### Raman/SERS Confirmation of Aptamer Immobilization and Ag-Assisted Enhancement

Thiol-modified dopamine aptamers were immobilized on printed gold nanoparticle surfaces. A 50 μL aliquot of 1 μM dopamine aptamer was drop-cast onto the surface for 2 h, followed by washing. Subsequently, 30 μL of 50 nm silver (Ag) nanoparticles was introduced to enhance surface-enhanced Raman scattering (SERS) through Ag–Au junctions and nanoscale “hot spots,” which amplify the local electromagnetic field. For comparison, SERS spectra were collected for bare gold (Au) nanoparticles (20, 40, and 80 nm) and Au with immobilized aptamers (Au + Apt). Each SERS spectrum represents an average of five measurements.

Raman/SERS spectra of bare Au nanoparticles (20, 40, and 80 nm) showed no distinct features in the 600–1700 cm^–1^ range. Whereas Au + Apt spectra displayed characteristic nucleic acid fingerprints due to the single-stranded DNA aptamer, exhibiting prominent SERS signatures of oligonucleotides (Figure 3b). Band assignments were determined based on previously reported Raman spectral analyses and are summarized in Table 1.^37–39^

**Table 1:** Assignment of the SERS Bands of the Aptamer

**ν (cm** ^**–**^ **^1^** **)**	Base	Vibration
∼718	A	Ring stretching
∼833	T	C–C and C–N stretching
∼1125	A	N–C
∼1236	T	Cycle stretching
∼1307	A/G	N–C
∼1443	G	N–C
∼1544	G	N–C
∼1590	G	*N* = C/ring

New and stronger DNA bands emerged at ∼718 cm^–1^ (adenine ring stretching) for 20 nm Au nanoparticles, with band intensities increasing with nanoparticle size (80 nm >40 nm >20 nm). The 80 nm Au + Apt configuration exhibited strong, distinct oligonucleotide peaks, as detailed in Table 1.

Overall, the Raman/SERS data confirm successful aptamer grafting onto printed Au layers and demonstrate size-dependent and Ag-assisted plasmonic enhancement. Also, Raman spectra of dopamine (DA), uric acid (UA), and ascorbic acid (AA) powders revealed strong, sharp bands characteristic of these molecules. (Figure S5a–c)

### Effect of Aptamer Immobilization and MCH Backfilling

Plasma-printed AuNP electrodes (20, 40, 80 nm) were exposed to 50 μL of 1 μM aptamer for 2 h, rinsed, and characterized by DPV in 25 μM dopamine before and after immobilization (Figure 4a–c). After immobilization, all electrodes showed a reproducible positive shift in the peak near +0.20 V, with modest reshaping of the current profile. The magnitude of ΔE varied with particle size, indicating a size-dependent interfacial response.

**Figure 4:**
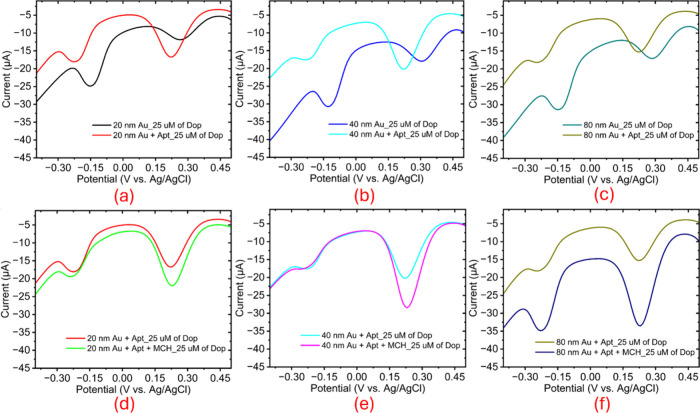
DPV responses of AuNP electrodes (20, 40, and 80 nm) in 25 μM dopamine (DA) before and after aptamer immobilization (a–c) and after MCH backfilling (d–f), showing size-dependent current variations and surface modification effects.

This shift is attributed to the formation of the aptamer monolayer on Au, altering interfacial potential and electron-transfer kinetics: the negatively charged phosphate backbone and steric barrier increase overpotential for dopamine redox, shifting peaks positively. Thus, peak displacement serves as an electrochemical signature of immobilization, with ΔE as a metric for surface coverage comparison. After aptamer immobilization, electrodes were treated with 1 mM MCH to passivate unbound Au sites and enhance surface order. DPV in 25 μM dopamine (Figure 4d–f) showed increased current intensity after MCH backfilling.

This enhancement indicates MCH improves aptamer layer orientation and packing, facilitating efficient charge transfer. Short-chain MCH likely aligns aptamers upright, reducing steric hindrance and enhancing dopamine accessibility. Variations in current enhancement across sizes suggest size-dependent interfacial effects, where surface curvature and binding sites influence molecular arrangement.

### Dopamine Sensitivity, Selectivity, and Repeatability

DPV measurements revealed distinct electrochemical responses across nanoparticle sizes. Dopamine exists in its reduced catechol form in solution, interfacial oxidation to dopamine-o-quinone occurs at the electrode surface under the applied potential, followed by electrochemical reduction back to dopamine, giving rise to the cathodic peak near –0.2 V. The systematic increase of this peak with dopamine concentration confirms its direct association with dopamine redox processes. DPV response observed near +0.2 V vs Ag/AgCl is attributed to intrinsic electrochemical activity of the AuNP layer, including gold surface states and Au–carbon interfacial charge transfer. This response is present even in the absence of dopamine and remains largely insensitive to dopamine concentration, indicating that it does not originate from dopamine electrochemistry. For 20 nm AuNPs with aptamer and MCH, DPV curves showed well-defined cathodic peak near −0.2 V, with current increasing from −5 μA at 0 μM to −35 μA at 100 μM DA, indicating a clear dose–response (Figure 5a,b). Similar concentration-dependent responses were observed for 40 nm (Figure S6) and 80 nm AuNPs (Figure S7).

**Figure 5:**
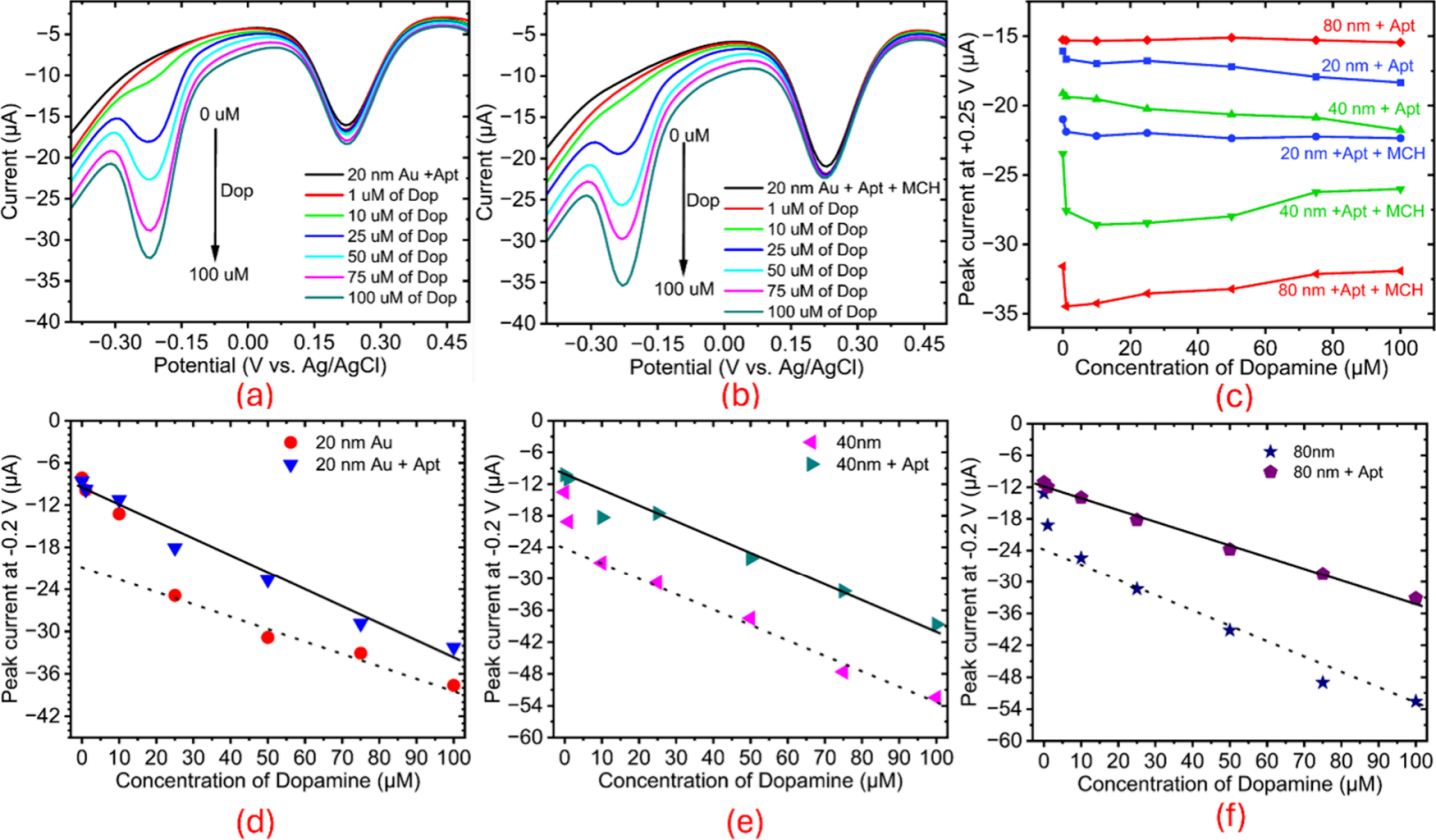
DPV Response and Sensitivity of Dopamine Sensors. (a, b) DPV scans of 20 nm Au + Apt and 20 nm Au + Apt + MCH electrodes in dopamine (DA) concentrations from 0 to 100 μM, showing well-defined oxidation peaks near −0.2 V with peak current increasing proportionally with [DA], c) Comparative DPV peak currents at +0.2 V across electrode configurations (20/40/80 nm Au + Apt, with and without MCH backfilling), d–f) Calibration plots and sensitivity slopes for 20, 40, and 80 nm electrodes, respectively.

Aptamer-immobilized electrodes exhibited improved linearity in differential pulse voltammetry (DPV) responses to increasing dopamine (DA) concentrations across the 1–100 μM range (Figure 5d–f). To start with, a blank screen-printed electrode (SPE) showed a negligible response to DA in the 1–100 μM range. For 20 nm AuNPs, sensitivity increases from 0.242 μA/μM with aptamer alone to 0.26 μA/μM with additional MCH backfilling, representing a 131% improvement over the blank SPE (from 0.11266 to 0.242 μA/μM) and a further 7.4% enhancement with MCH. For 40 nm AuNPs, aptamer immobilization yields 0.31 μA/μM, a 175% increase over the blank, though MCH backfilling reduces sensitivity to 0.236 μA/μM, a 23.9% decrease from the aptamer-only value, possibly due to surface saturation effects. For 80 nm AuNPs, sensitivity with aptamer alone is 0.216 μA/μM (92% above the blank), rising to 0.262 μA/μM with MCH, a 21.3% increase, suggesting optimized aptamer orientation.

These trends highlight a size-dependent response, with 40 nm AuNPs exhibiting the highest aptamer-only sensitivity (0.31 μA/μM) (Figure 6a), while MCH backfilling consistently refines performance, most notably for 80 nm (21.3% gain) and 20 nm (7.4% gain). The data underscores the role of nanoparticle size and MCH in tailoring the electroactive surface for DA detection. DPV peak currents at +0.2 V increase with AuNP size (80 nm >40 nm >20 nm), both with and without MCH backfilling, is attributed to the higher electroactive surface area and enhanced interfacial charge-transfer characteristics of larger AuNP layers. MCH consistently enhances signal clarity and reproducibility across all sizes (Figure 6c). Similarly, the peak currents observed for 40 and 80 nm AuNP-based electrodes at +0.2 V with the corresponding DPV data presented in the Supporting Information (Figures S6 and S7).

**Figure 6:**
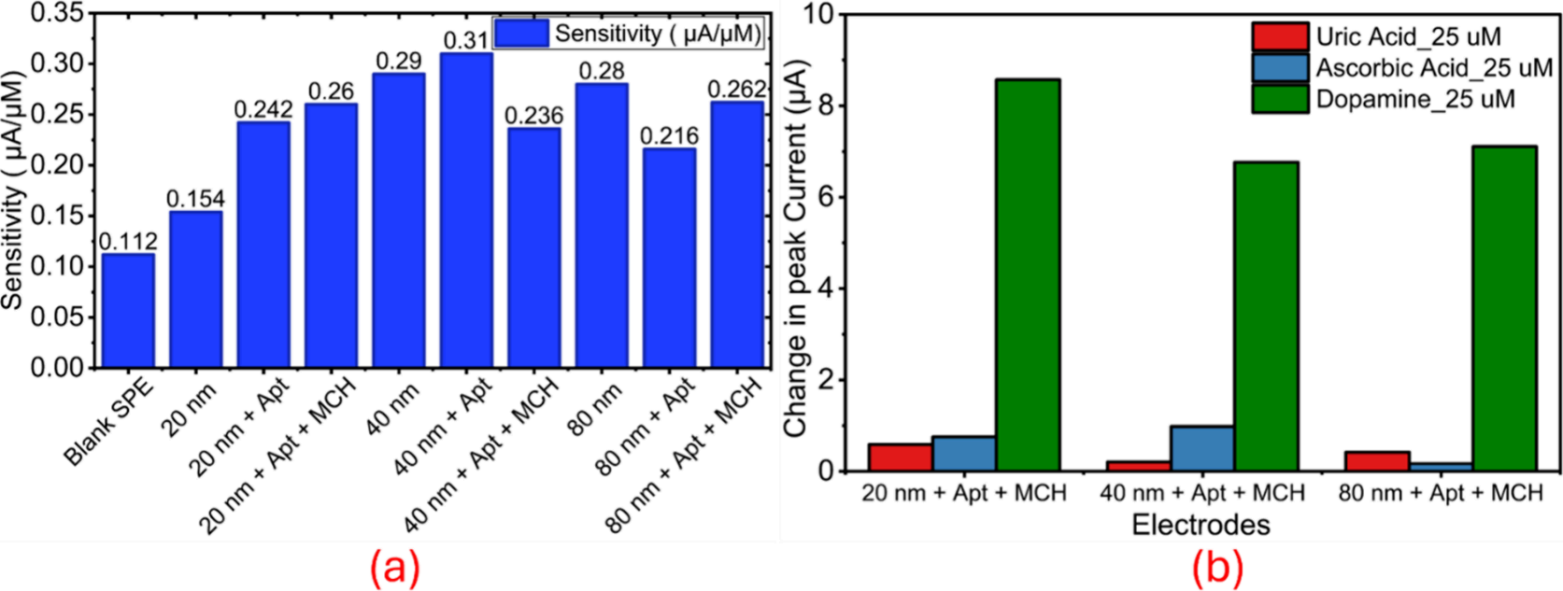
Sensitivity and selectivity analysis of plasma-printed AuNP-based dopamine sensors, a) Sensitivity comparison of blank SPE and AuNP electrodes (20, 40, 80 nm) before and after aptamer immobilization and MCH backfilling, showing size-dependent variations in μA/μM response, b) Change in peak current (ΔI) at – 0.25 V for 25 μM dopamine (DA), uric acid (UA), and ascorbic acid (AA), confirming high selectivity of the aptamer-functionalized electrodes toward DA.

The dependence of dopamine (DA) sensitivity on the morphology of the plasma-printed structures was clearly evident. The 40 nm electrodes exhibited the highest DA sensitivity, followed by the 20 and 80 nm counterparts. This can also be observed in Figure 5f, where the anodic peak current (I_p_) for the 80 nm electrodes was markedly lower than that of the 20 and 40 nm electrodes, consistent with their coarser surface morphology and reduced electroactive area. The enhanced performance of the 40 nm electrodes can be attributed to an optimal balance between nanoparticle packing density and surface conductivity sufficiently small to sustain a high electroactive surface area, yet large enough to form a mechanically stable structure. In contrast, excessive agglomeration of 80 nm particles during plasma-assisted printing leads to heterogeneous layer growth, reduced charge transport, and broadened redox features, thereby diminishing DA oxidation efficiency.

The selectivity of the aptamer-functionalized electrodes was evaluated using 25 μM each of dopamine (DA), uric acid (UA), and ascorbic acid (AA) (Figure S5). All configurations exhibited a strong positive change in peak current (ΔI ≈ 6–9 μA) for DA, while UA and AA elicited negligible responses (ΔI < 1 μA), confirming excellent specificity (Figure 6b). Overall, maintaining nanoscale integrity and uniform particle distribution is essential, as electrodes printed using 20 and 40 nm colloidal AuNP solutions at higher plasma voltages (18 kV) produced stable, highly electroactive layers on carbon substrates, exhibiting exceptional structural robustness and consistent redox performance toward dopamine detection.

## Conclusions

This work demonstrates the effective use of plasma-assisted printing to fabricate gold nanoparticle (AuNP)-based electrochemical sensors specifically for dopamine detection on commercial screen-printed carbon electrodes. The binder-free, low-temperature process enables direct deposition of AuNP suspensions without surfactants, wet chemistry steps, or thermal curing, while the plasma simultaneously activates the substrate surface to create nanoscale pores and active sites that provide strong nanoparticle anchoring. This surface modification was essential for obtaining uniform, well-adhered AuNP layers with excellent electrochemical stability during repeated measurements. By systematically studying AuNP sizes (20, 40, and 80 nm) and plasma voltages (14–18 kV), the work establishes how plasma conditions and nanoparticle dimensions influence layer morphology, cluster formation, and electron-transfer behavior relevant to dopamine sensing.

Among the fabricated electrodes, 40 nm AuNP layers printed at 18 kV delivered the most balanced dopamine sensing performance, while 20 nm layers showed faster electron-transfer kinetics and 80 nm layers exhibited reduced activity due to agglomeration. Integration of thiolated dopamine aptamer immobilization with MCH backfilling significantly improved sensitivity, selectivity against ascorbic acid and uric acid, and linear detection across the 1–100 μM range. The printed layers also preserved the nanoscale and optical characteristics of the precursor nanoparticles after plasma deposition. This study presents a scalable, reliable strategy for developing stable, size-tunable plasma-printed AuNP electrodes tailored for selective dopamine electrochemical detection on disposable sensing platforms.

## Supplementary Material



## Notes

SEM images of AuNP layers at 14 kV and 18 kV; EDS elemental mapping; UV–Vis spectra of AuNP solutions and printed layers; Raman/SERS spectra of aptamer-modified AuNP surfaces and analytes; DPV responses and calibration plots for 40 and 80 nm electrodes; selectivity studies with ascorbic acid and uric acid; additional electrochemical cycling stability data (PDF)
