# Supplementary material for: Size-Dependent Electrochemical Response and Dopamine Sensitivity of Aptamer-Modified Printed Gold Nanoparticle Structures
Source: ACS Omega. 2026 May 7;11(19):28255–64. doi: 10.1021/acsomega.5c13417 (PMC13191519; doi:10.1021/acsomega.5c13417)
Supplement: Supplementary file 1 [file ao5c13417_si_001.pdf]

Supporting information

Size-Dependent Electrochemical Response and Dopamine Sensitivity of Aptamer-Modified Printed Gold Nanoparticle Structures

Santhosh Adhinarayanan[1], Harikrishnan Muraleedharan Jalajamony[2], Soumadeep De[2], Renny Edwin Fernandez[1]*

[1] Department of Engineering, Norfolk State University, Norfolk, VA 23504, USA

[2] Department of Materials Science and Engineering, Norfolk State University, Norfolk, VA 23504, USA

* Corresponding author- Email: refernandez@nsu.edu

1. SEM Analysis (Extended Version)

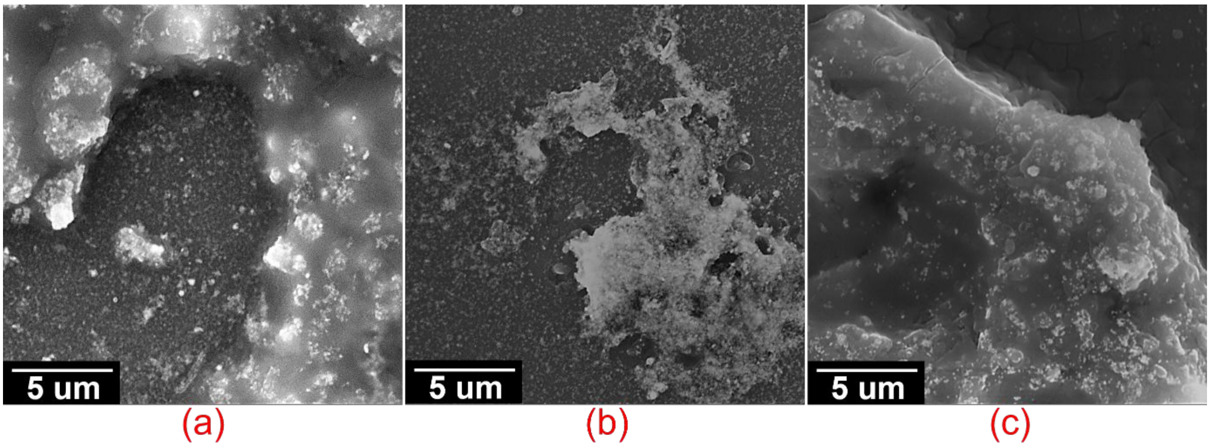

Fig. S1. SEM micrographs of plasma-printed AuNP samples at 14kV: a) 20 nm AuNP ink, b) 40 nm AuNP ink, c) 80nm AuNP ink, showing agglomerated nanoparticle cluster formation due to insufficient plasma activation and retained NaCl residues.

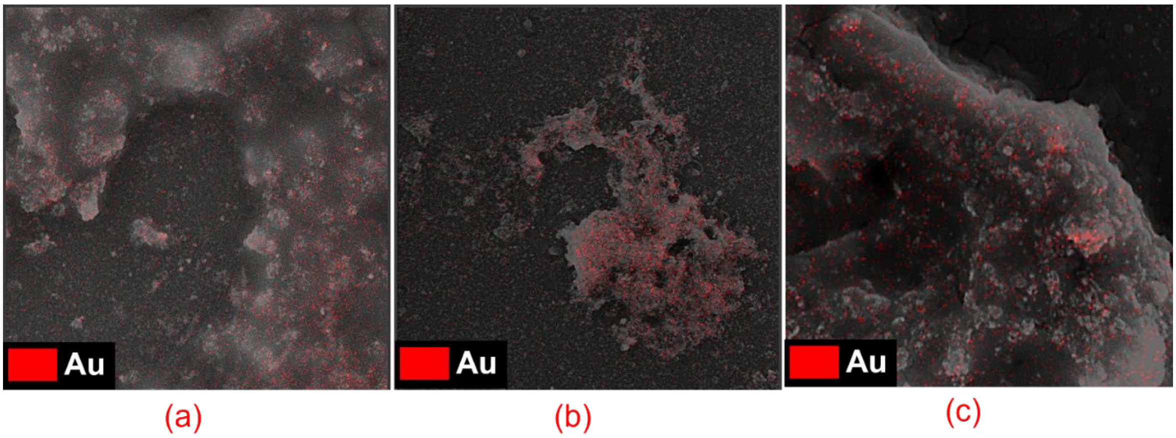

Fig. S2. EDS elemental mapping (acquired in SEM at 14 kV) of plasma-printed AuNP films from a) 20 nm, b) 40 nm, and c) 80 nm inks. Red pixels indicate the elemental distribution of Au.

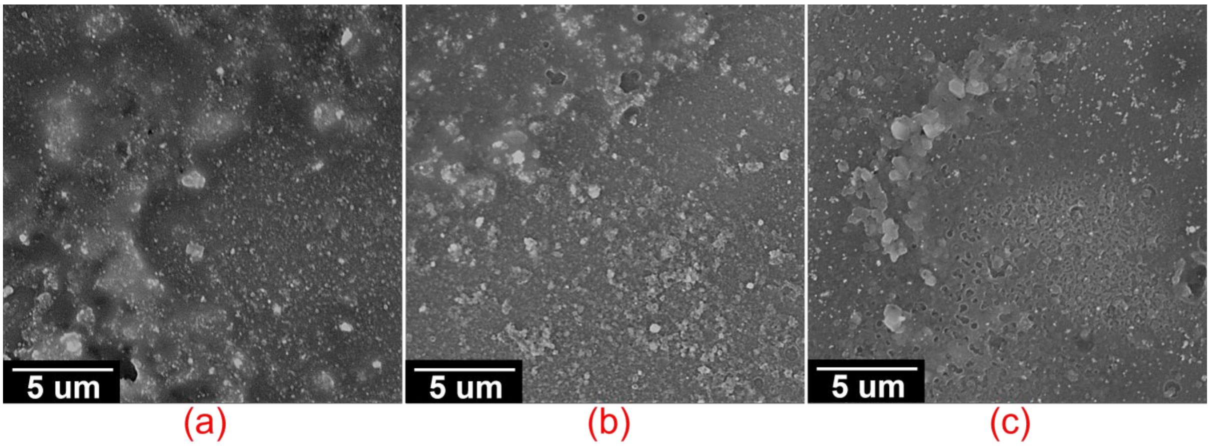

Fig. S3. SEM micrographs of plasma-printed AuNP samples at 14kV: a) 20 nm AuNP ink, b) 40 nm AuNP ink, c) 80nm AuNP ink, highlighting the significant reduction in aggregate size at the higher plasma voltage

2. Optical Characterization (Extended Version)

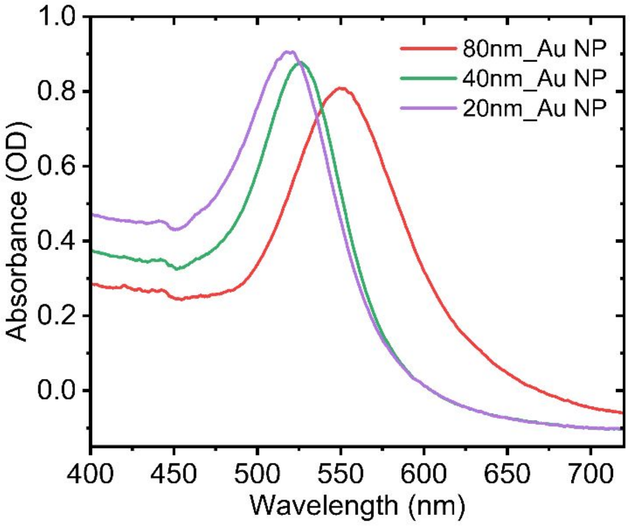

Fig. S4. UV–Vi's spectra of colloidal AuNPs (20, 40, and 80 nm) showing distinct LSPR peaks.

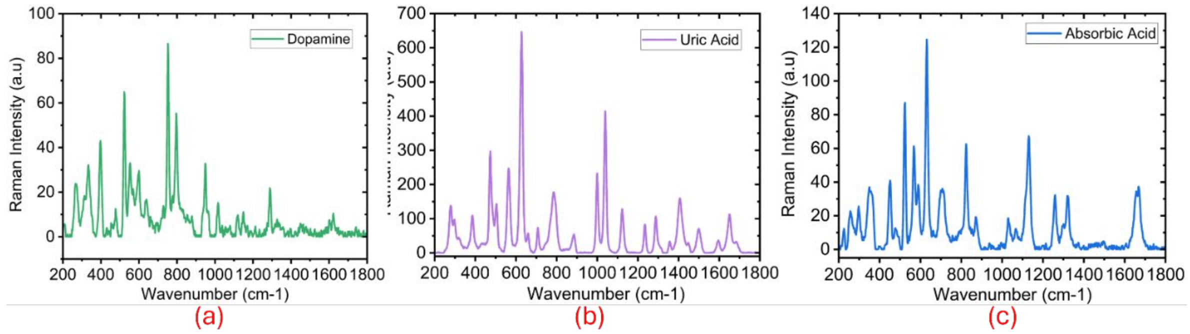

Fig. S5. Raman spectra of a) dopamine, b) uric acid, and c) ascorbic acid powders recorded in the 200–1800 cm^{-1} range, showing their characteristic molecular vibration bands.

3. Sensitivity and Selectivity Analysis (Extended Version)

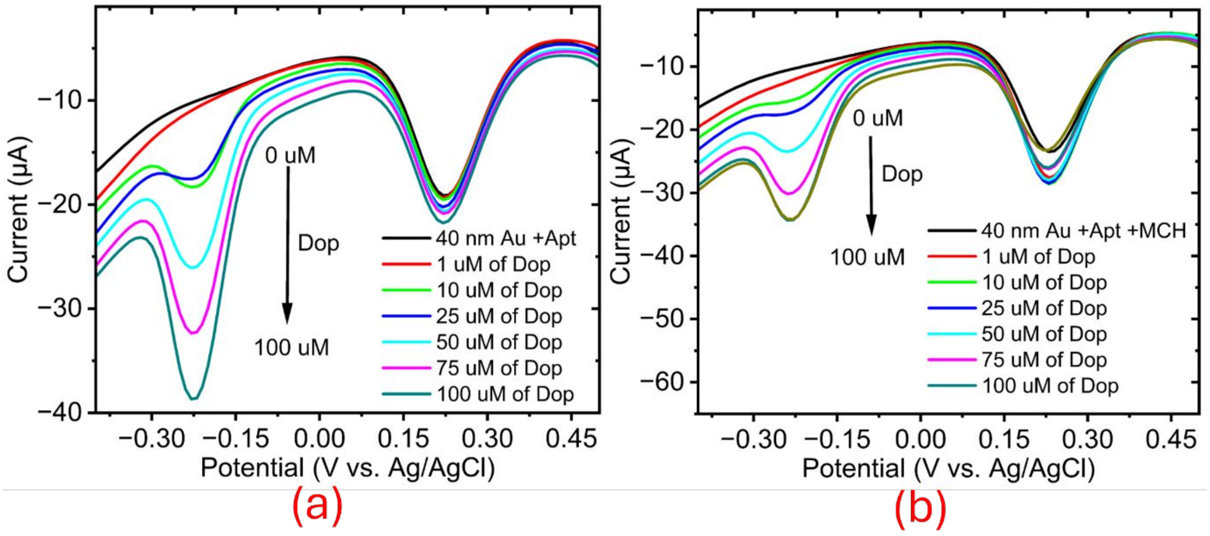

Fig. S6. DPV response of 40 nm AuNP-based dopamine sensors at different dopamine (DA) concentrations (0–100 μM):(a) DPV scans recorded using 40 nm Au + aptamer (Apt) electrodes, and(b) DPV scans recorded using 40 nm Au + Apt + MCH electrodes, showing well-defined dopamine-related peaks near −0.2 V vs. Ag/AgCl with peak current increasing proportionally with [DA]

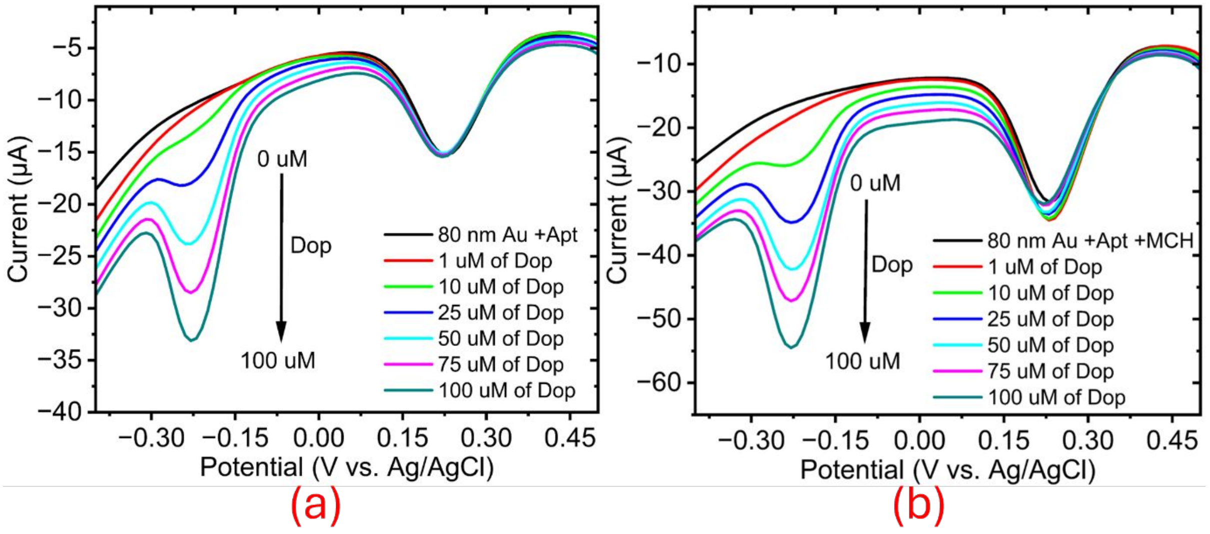

Fig. S7. DPV response of 80 nm AuNP-based dopamine sensors at different dopamine (DA) concentrations (0–100 μM):(a) DPV scans recorded using 80 nm Au + aptamer (Apt) electrodes, and(b) DPV scans recorded using 80 nm Au + Apt + MCH electrodes, showing well-defined dopamine-related peaks near −0.2 V vs. Ag/AgCl with peak current increasing proportionally with [DA]

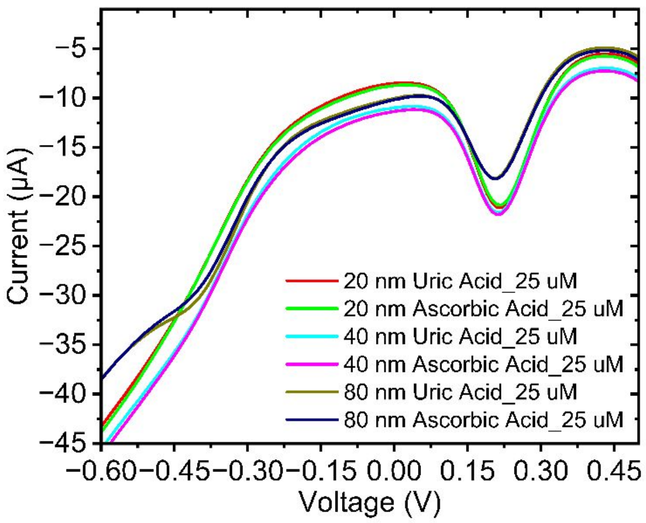

Fig. S8. Differential pulse voltammetry (DPV) responses of plasma-printed AuNP electrodes (20, 40, 80 nm) recorded in 25 μM uric acid (UA) and ascorbic acid (AA) solutions. The overlapping curves indicate negligible oxidation response for UA and AA compared to dopamine, confirming the high selectivity of the aptamer-functionalized electrodes toward dopamine.
